# Historical contributions and future horizons of Japanese orthopaedic oncology: from surgical innovation to nationwide collaborations

**DOI:** 10.1007/s00264-026-06999-6

**Published:** 2026-09-02

**Authors:** Akira Kawai, Keisuke Horiuchi, Koji Hiraoka, Toshifumi Ozaki, Kazuhiro Tanaka, Hirotaka Kawano

**Affiliations:** 1 Department of Musculoskeletal Oncology and Rehabilitation Medicine, National Cancer Center Hospital, Tokyo, Japan; 2 Department of Rehabilitation Medicine, Kanagawa Cancer Center, Kanagawa, Japan; 3 Department of Orthopaedic Surgery, National Defense Medical College, Saitama, Japan; 4 Department of Orthopaedic Surgery, Kurume University, Kurume, Japan; 5 Department of Orthopaedic Surgery, Okayama University Graduate School of Medicine, Dentistry and Pharmaceutical Sciences, Okayama, Japan; 6 Department of Orthopaedic Surgery, Oita University, Oita, Japan; 7 Department of Orthopaedic Surgery, Teikyo University School of Medicine, Tokyo, Japan

**Keywords:** Orthopaedic oncology, JBSTTR, JMOG, JCOG-BSTTSG, JSTAR, Onco-orthopaedics

## Abstract

**Purpose:**

Bone and soft tissue sarcomas are rare, heterogeneous malignancies presenting major diagnostic and therapeutic challenges. Over the past century, Japanese orthopaedic surgeons have occupied a unique position as both primary surgeons and comprehensive oncologists. On the occasion of the Centennial of the Japanese Orthopaedic Association (JOA), this review synthesizes the key historical milestones and future prospects of Japanese orthopaedic oncology.

**Methods:**

A narrative synthesis was structured across five consolidated domains: (1) Surgical Innovation; (2) Advanced Basic Research and Novel Therapeutics; (3) The Japan Bone and Soft Tissue Tumor Registry (JBSTTR); (4) Nationwide Collaborative Research Groups and Multidisciplinary Platforms; and (5) Onco-Orthopaedics and Future Musculoskeletal Care.

**Results:**

Japanese orthopaedic oncologists established quantitative surgical margin concepts, pioneered Total En Bloc Spondylectomy (TES) for spinal tumours, and developed recycled treated autografts (freezing, pasteurization, and irradiation). Translational research milestones encompass caffeine-potentiated chemotherapy, peptide-based immunotherapy, oncolytic viral therapies, Japan’s first investigator-initiated clinical trial in oncology, and carbon-ion radiotherapy. Furthermore, a robust nationwide infrastructure has been forged through six decades of the JBSTTR alongside collaborative research engines—the Japanese Musculoskeletal Oncology Group (JMOG), the Japan Clinical Oncology Group Bone and Soft Tissue Tumor Study Group (JCOG-BSTTSG), and the Japan Ewing Sarcoma Study Group (JESS)—and the multidisciplinary platform Japanese Association of Sarcoma Treatment and Research (JSTAR). Expanding beyond primary sarcoma care, the conceptual framework of Onco-Orthopaedics has pioneered a holistic paradigm for managing locomotive syndrome in cancer patients "Cancer Locomo".

**Conclusion:**

The surgeon-led collaborative ecosystem in Japan bridges surgical precision, translational science, and nationwide multidisciplinary platforms, offering a sustainable model for advancing musculoskeletal oncology.

## Introduction

Bone and soft tissue sarcomas are rare, clinically diverse, and pathologically complex malignancies accounting for less than 1% of all human cancers [1]. Managing these neoplasms presents severe global obstacles, as single institutions cannot independently accumulate sufficient case volumes to generate high-level clinical evidence.

**Fig. 1:**
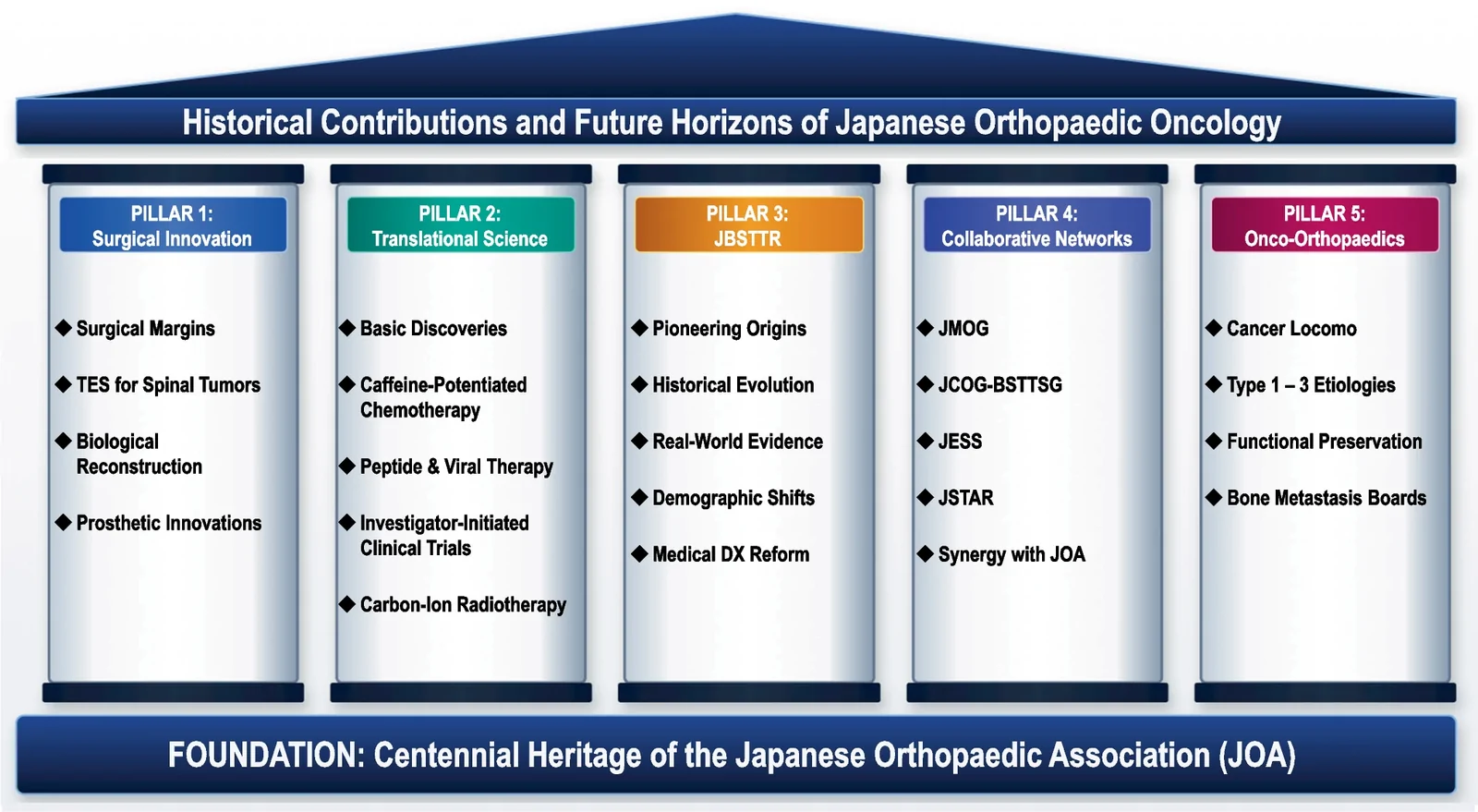
Structural architectural model of the five pillars of Japanese orthopaedic oncology. Pillar 1 (Surgical Innovation): Quantitative surgical margins, Total En Bloc Spondylectomy (TES) for spinal tumors, biological reconstruction using recycled autografts (freezing, pasteurization, irradiation), and prosthetic innovations (constrained hip implants, iodine coating). Pillar 2 (Translational Science): Basic genetic/molecular discoveries, caffeine-potentiated chemotherapy, peptide/viral therapies, investigator-initiated clinical trials, and carbon-ion radiotherapy. Pillar 3 (JBSTTR): Six-decade nationwide registry evolution, Red/Blue book archives, real-world evidence, demographic shifts, and electronic DX transformation. Pillar 4 (Collaborative Networks): Nationwide study groups (JMOG, JCOG-BSTTSG, JESS), multidisciplinary platforms (JSTAR), and synergy with the JOA. Pillar 5 (Onco-Orthopaedics): Management of Cancer Locomo (Types 1–3 aetiologies), functional preservation, and multidisciplinary bone metastasis boards

In Japan, orthopaedic surgeons have historically occupied a distinctive, comprehensive role. Unlike care models divided among separate medical and surgical subspecialties, Japanese orthopaedic oncologists serve as primary providers—directing surgical extirpation, complex limb reconstruction, perioperative systemic therapy, and long-term surveillance. On the occasion of the Centennial of the Japanese Orthopaedic Association (JOA), this review synthesizes the historical contributions and future horizons of Japanese orthopaedic oncology across five core pillars (Fig. 1).

## PILLAR 1: Surgical innovation

The management of musculoskeletal sarcomas underwent a historic paradigm shift in the late 20th century, transitioning from mandatory amputation to limb-salvage procedures. Japanese orthopaedic surgeons played a pivotal role in establishing the oncological and surgical foundations for safe, function-preserving resections and biological reconstructions.

### Quantitative surgical margin concepts

Limb-sparing surgery relies on achieving adequate surgical margins to prevent local recurrence while preserving critical neurovascular structures. While Enneking’s landmark surgical staging system established the qualitative classification of margins (intralesional, marginal, wide, and radical) [2], its practical application often suffered from inter-observer variability, particularly in complex anatomical locations with tight anatomical boundaries.

To eliminate this ambiguity, Dr. Noriyoshi Kawaguchi and colleagues at the Cancer Institute Hospital introduced the concept of quantitative margin evaluation [3]. Anatomical structures were systematically categorized based on their inherent resistance to tumour invasion: thick barriers (e.g., major deep fascia, joint capsules, periosteum, and un-eroded cartilage) and thin barriers (e.g., muscle epimysium, perineurium, or vessel adventitia). By integrating physical measurement with the barrier quality index, surgeons could calculate a standardized "quantitative margin."

Correlating these margin scores with local recurrence outcomes demonstrated that a quantitative margin exceeding 2 cm provided local control rates comparable to classical radical resections. Formally adopted by the JOA Musculoskeletal Tumor Committee [4], this objective scoring framework transformed pre-operative surgical planning and post-operative surgical quality assessment, providing a universally reproducible standard in orthopaedic oncology.

### Total En Bloc Spondylectomy (TES) for spinal tumours

Primary spinal sarcomas and solitary vertebral metastases historically carried a dismal prognosis due to anatomical complexity and proximity to the spinal cord. Intralesional piecemeal excision frequently resulted in tumor seeding and local failure. To overcome these limitations, Prof. Katsuro Tomita and colleagues at Kanazawa University developed Total En Bloc Spondylectomy (TES) to achieve complete, margin-free wide resection by removing the affected vertebra as a single contiguous mass [5]. Utilizing flexible thread saws (T-saws) for posterior laminar transection followed by en bloc extirpation of the vertebral body and rigid circumferential reconstruction, TES revolutionized the oncological management of primary spinal malignancies and select solitary metastases worldwide [6–8].

### Biological reconstruction using treated autografts

Massive endoprostheses and bone allografts are often associated with serious long-term mechanical and biological complications, including loosening, structural failure, wear, and infection in young long-term survivors [9]. To provide a durable alternative, Japanese orthopaedic oncologists pioneered biological reconstruction using recycled, tumour-bearing autografts [10].

This concept utilizes the patient’s own resected tumour-bearing bone, which is treated ex vivo to eradicate all viable tumour cells while preserving its anatomical shape, structural congruence, and osteoconductive potential. After tumour devitalisation, the recycled autograft is reimplanted and secured with rigid internal fixation, allowing gradual host-donor bone union and long-term biological remodeling. Japanese investigators standardized three primary devitalisation modalities:

- **Freezing in liquid nitrogen:** Immersion in liquid nitrogen (−196°C) for 20 minutes induces total cell necrosis while preserving structural strength. Reconstructions using liquid nitrogen-treated autografts have demonstrated excellent long-term clinical and radiological outcomes, particularly in intercalary reconstructions where the adjacent major joint (e.g., hip, knee, ankle, or shoulder) is preserved [11, 12]. Pedicled freezing techniques (freezing the bone segment in situ while preserving soft-tissue bridges) further accelerate revascularization [13].
- **Pasteurization:** Heating resected bone in saline at 60°C–65°C for 30 minutes eradicates tumour cells while preserving essential bone morphogenetic proteins better than autoclaving [14, 15].
- **Extracorporeal irradiation:** Exposing the resected bone segment to a single high dose of irradiation (typically 50 Gy) ex vivo prior to reimplantation provides reliable, uniform tumour eradication [16, 17].

**Fig. 2:**
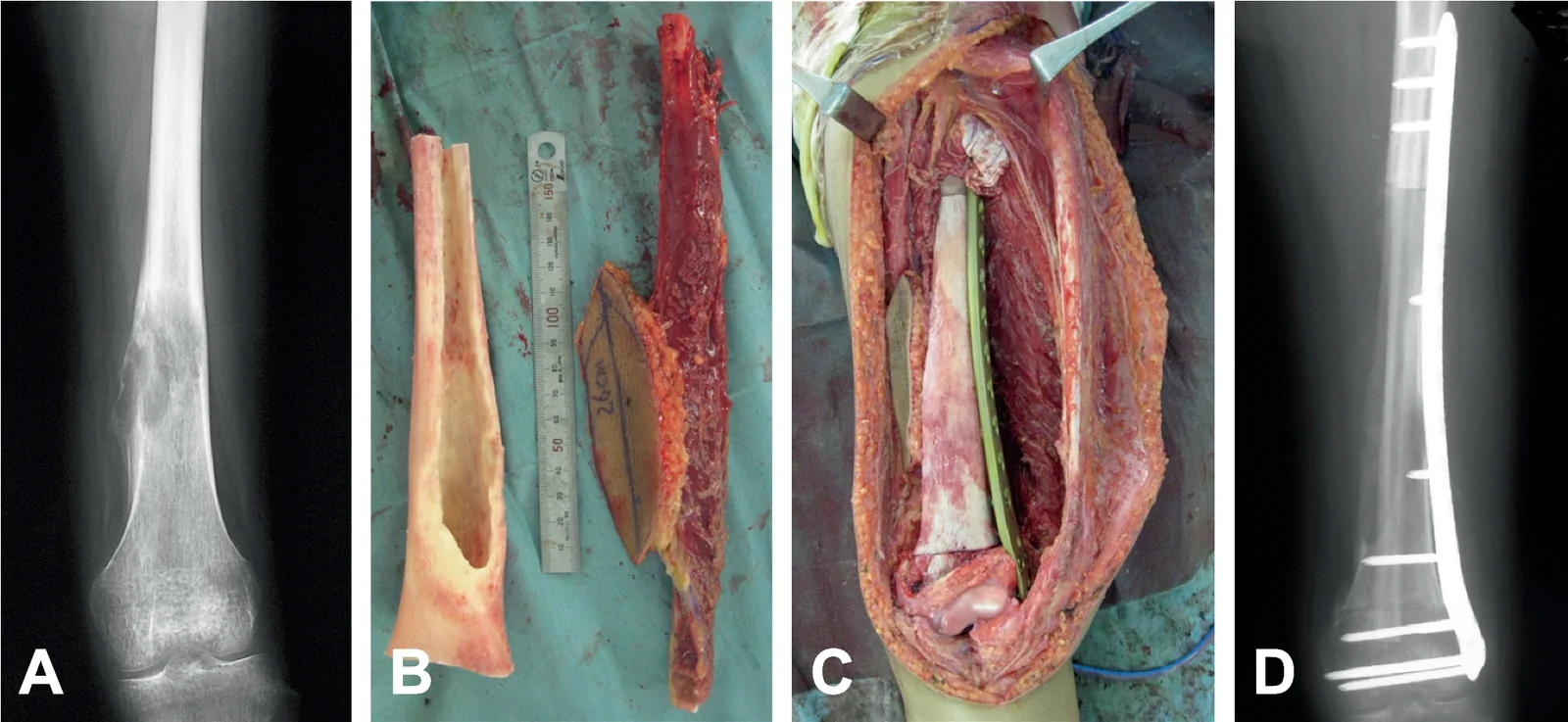
Biological reconstruction using a liquid nitrogen-treated recycled autograft combined with a free vascularized fibular graft (osteosarcoma of the left distal femur, 17-year-old female). **A** Pre-operative anteroposterior radiograph showing a bone-destructive lesion in the distal femoral diaphysis and metaphysis. **B** Gross intra-operative photograph of the resected tumor-bearing distal femur devitalized in liquid nitrogen (−196°C) (left) alongside the harvested free vascularized fibular graft (right). **C** Intra-operative photograph demonstrating composite biological reconstruction: the liquid nitrogen-treated recycled autograft is reinforced with the intramedullary vascularized fibular graft and secured with a rigid long locking plate. **D** Post-operative anteroposterior radiograph at 4-year follow-up showing solid host-donor junctional bone union, stable implant fixation, and excellent limb preservation without local recurrence.

The greatest advantages of these biological autograft techniques are perfect anatomical matching, preservation of joint capsules and ligamentous attachments, and the potential for permanent, lifelong osteointegration without foreign body reactions. Because recycled autografts act as a devitalized scaffold, host-donor junctional bone union typically requires prolonged time (often 6 to 12 months or longer, particularly under perioperative chemotherapy) [18]. To address this, Japanese surgeons frequently combine treated autografts with supplementary vascularized bone grafts (such as free vascularized fibular grafts) or robust plating, achieving high ultimate union rates and exceptional long-term limb function (Fig. 2) [19].

### Prosthetic innovations

In addition to biological reconstructions, Japanese orthopaedic surgeons developed specialized endoprosthetic innovations. To prevent post-operative dislocation—a major drawback of extensive proximal femoral or acetabular resections—Prof. Atsumasa Uchida at Osaka University developed a novel, constrained (controlled) tumour endoprosthetic system [20]. Although constrained tumour endoprostheses carry a recognized risk of long-term mechanical complications and infection, novel pelvic implants designed to mitigate these failure modes are actively under development.

Furthermore, driven primarily by research at Kanazawa University, antibacterial iodine-supported titanium implants were developed and recently achieved regulatory approval in Japan [21–23]. By releasing elemental iodine locally to suppress periprosthetic infection without systemic toxicity or compromising bone ingrowth, this technological breakthrough holds immense promise for broader clinical application and validation across immunocompromised oncology populations.

## PILLAR 2: Advanced basic research and novel therapeutics

Bridging clinical practice with molecular biology and innovative therapeutics, Japanese orthopaedic scientists have driven major breakthroughs from bench to bedside.

### Basic discoveries

Japanese researchers contributed significantly to deciphering sarcoma biology. Landmark studies led by Prof. Junya Toguchida and his team at Kyoto University provided critical breakthroughs in understanding genetic alterations and allele-specific imprinting of the *RB1* gene [24, 25], as well as germline *TP53* mutations in osteosarcomas [26–28]. Another landmark collaborative study established that the t(X;18)(p11;q11) translocation generating *SS18-SSX1* or *SS18-SSX2* fusion transcripts is a defining biological and diagnostic hallmark of synovial sarcoma [29–31]. Collaborative multi-omics analyses further characterized distinct genomic, epigenomic, and proteomic characteristics across bone and soft tissue sarcomas, including liposarcomas and myxofibrosarcomas [32–34].

### Caffeine-potentiated chemotherapy

Pioneered by Prof. Hiroaki Tsuchiya and colleagues at Kanazawa University, caffeine-potentiated chemotherapy represents a landmark Japanese contribution to pharmacological chemosensitization in musculoskeletal oncology [35, 36]. The strategy utilizes caffeine to abrogate the G2/M cell-cycle checkpoint induced by DNA-damaging agents (such as doxorubicin, cisplatin, and ifosfamide), overriding cell-cycle arrest and forcing DNA-damaged sarcoma cells into premature mitotic catastrophe and apoptosis [37].

In clinical application, pre-operative caffeine administration combined with chemotherapy achieved remarkably high histological response rates (>90% tumour necrosis) across osteosarcomas and high-grade soft tissue sarcomas. This profound histological response allowed surgeons to perform intentional marginal resections adjacent to critical neurovascular bundles, successfully achieving high limb-preservation rates without compromising local control or overall survival [38, 39].

### Peptide-based immunotherapy & oncolytic viral therapy

Early translational immunotherapy was pioneered at Sapporo Medical University, where researchers developed tumour-antigen-specific peptide vaccines targeting cytotoxic T-lymphocyte epitopes, such as *SYT-SSX* breakpoint peptides in synovial sarcoma and papillomavirus binding factor (PBF) in osteosarcoma [40, 41]. Early-phase clinical trials demonstrated that the administration of SYT-SSX- or PBF-derived peptides—particularly when combined with incomplete Freund's adjuvant and interferon-alpha—successfully induced antigen-specific cytotoxic T-lymphocyte responses and achieved disease stabilization or partial tumour regression in advanced or metastatic cases [42, 43].

Researchers at Kagoshima University advanced survivin-responsive conditionally replicating adenoviruses that selectively lyse survivin-overexpressing sarcoma cells [44]. These oncolytic viruses selectively replicate within and lyse survivin-expressing cancer cells while sparing surrounding normal tissues, offering a highly targeted, potent precision approach that has advanced into early clinical evaluation for refractory tumours [45].

### Investigator-initiated clinical trials and novel chemotherapeutic agents

Japanese orthopaedic networks led a historic trial evaluating imatinib mesylate in relapsed or refractory KIT-positive or PDGFR-positive sarcomas [46]. While the trial did not meet its primary endpoint, it holds monumental significance as Japan's very first investigator-initiated clinical trial for anticancer therapeutics under the revised Pharmaceutical Affairs Law. This pioneer trial established the regulatory, ethical, and operational framework required for academic drug development in Japan. Leveraging these established frameworks, Japanese orthopaedic groups subsequently led pivotal multi-center trials that secured regulatory approvals for novel systemic agents for soft tissue sarcomas, including pazopanib [47, 48], trabectedin [49–53], and eribulin [54, 55]. Approved in the 2010 s, these agents have now become globally recognized standard second-line and subsequent therapies for advanced and metastatic soft tissue sarcomas [56, 57].

### Carbon-ion radiotherapy

In collaboration with the National Institute of Radiological Sciences, Japanese orthopaedic oncologists established carbon-ion radiotherapy as a definitive curative modality for unresectable bone and soft tissue sarcomas [58–60]. Providing superior dose localization and high biological effectiveness, carbon-ion therapy achieves outstanding local control and overall survival rates, offering a highly effective non-surgical option—particularly for unresectable sacral chordomas and spinal sarcomas [61, 62].

## PILLAR 3: The Japan Bone and Soft Tissue Tumor Registry (JBSTTR)

The central pillar supporting clinical, epidemiological, and scientific progress of musculoskeletal oncology in Japan is the Japan Bone and Soft Tissue Tumor Registry (JBSTTR). Operating continuously for six decades, it is one of the world's longest-running tumor-specific registries.

### Pioneering origins and historical evolution

The conceptual foundation of nationwide registration in Japan was laid in the early 1950 s by Prof. Tamikazu Amako at Kyushu University, who initiated a regional registry for bone tumours in western Japan. Following the establishment of the National Cancer Center (NCC) in Tokyo in 1962, Dr. Iwao Maeyama—the founding chief of the Department of Orthopaedic Surgery—expanded this platform into a national registry, a mission subsequently sustained and refined by his successor, Dr. Hisatoshi Fukuma. In 1964, through a formal partnership between the NCC and the Bone Tumor Committee of the JOA, the national bone tumour registry was officially launched, followed by the incorporation of malignant soft tissue tumours in 1985.

For decades, data collection relied on annual paper-based submissions compiled manually into registry summaries—known as the "Red Books" (bone tumours) [63], and "Blue Books" (soft tissue tumors) [64]. A major structural modernization occurred in 2006 under the direction of Dr. Akira Kawai, when the JBSTTR transitioned to an electronic application system backed by institutional review board (IRB) approvals from both the NCC and the JOA. This reform enabled systematic five and ten year prospective follow-up tracking across accredited medical centers nationwide, establishing a highly granular database [65].

### Real-world evidence and future perspectives

Since opening JBSTTR data for secondary research proposals in 2013, the registry has powered dozens of peer-reviewed scientific studies, transforming raw registry data into clinical evidence [66]:

- **Epidemiological profile & demographic shifts:** Registry analyses encompassing thousands of primary bone and soft tissue sarcomas have established benchmark epidemiological profiles in Japan [67, 68]. Crucially, longitudinal registry tracking over 40 years revealed a shifting demographic landscape, demonstrating a noticeable increase in osteosarcoma incidence among middle-aged and elderly populations—a unique trend shifting from a classical unimodal distribution centered on adolescents toward a bimodal distribution with a second peak in older adults [69]. Longitudinal registry data also documented a paradigm shift toward conservative, non-surgical management for desmoid-type fibromatosis [70].
- **Granular big data and subgroup analyses:** By aggregating rare cases nationwide, the registry enabled detailed evaluation of elderly osteosarcoma patients [71], pelvic osteosarcoma management [72], ultra-rare malignancies such as alveolar soft part sarcoma and clear cell sarcoma [73, 74], and other characteristic findings of bone and soft tissue tumours [75–82]. Additionally, registry data enabled the development of the first Asian prognostic nomogram for extremity and trunk soft tissue sarcomas [83], enhancing individualized risk prediction.
- **Surgical quality assessment:** Leveraging advanced statistical techniques to control for confounding biases, registry-based studies evaluated real-world surgical practices [84]. Notably, propensity score-matched analyses using the JBSTTR data demonstrated that while unplanned excisions (UE) followed by secondary re-excision achieved overall survival rates comparable to primary planned excisions (PE), UEs posed substantial clinical disadvantages, significantly increasing the need for extensive secondary surgical procedures, including complex soft-tissue reconstructions [85].

Despite its successes, the JBSTTR faces contemporary operational challenges, including the heavy administrative burden placed on voluntary participating clinicians, data completeness, and follow-up attrition. To address these hurdles, the registry is undergoing a digital transformation. Initiatives are underway to transition from standalone software to secure online cloud platforms integrated directly with hospital Electronic Health Record systems. This automation aims to enable direct data extraction, drastically reduce manual data entry, and minimize missing data.

## PILLAR 4: Nationwide collaborative research groups and multidisciplinary platforms

Overcoming intrinsic obstacles of musculoskeletal oncology has necessitated nationwide collaboration, strategic harmonization, and the evolution of specialized, multi-institutional research networks across Japan.

### Nationwide collaborative research groups: JMOG, JCOG-BSTTSG, and JESS

To systematically address research questions ranging from cohort-based practice evaluations to high-grade prospective interventional trials, Japanese orthopaedic oncology established synergistic collaborative research engines:

#### Japanese Musculoskeletal Oncology Group (JMOG)

Founded in 1981 by Prof. Akio Tateishi at Teikyo University and incorporated as a non-profit organization in 2011 under the leadership of Dr. Takafumi Ueda at National Hospital Organization Osaka National Hospital, JMOG encompasses approximately 250 specialized investigators. Under successive leadership, including Prof. Hirotaka Kawano at Teikyo University, JMOG focuses on nationwide retrospective cohort studies, prospective observational registries, and surgical outcome analyses. Its scientific output spans premier peer-reviewed journals addressing critical clinical questions such as:

- rare entity profiles, including Ewing sarcoma [86], clear cell sarcoma of tendons and aponeuroses [87], and clear cell chondrosarcoma [88, 89],
- prognostic impact of the timing of lung metastasis onset in osteosarcoma [90],
- surgical procedures and implant-related outcomes [18, 91–94],
- prospective evaluations of symptomatic venous thromboembolism in sarcoma patients [95].

#### JCOG Bone and Soft Tissue Tumor Study Group (JCOG-BSTTSG)

Established in 2002 within the Japan Clinical Oncology Group (JCOG) under founding leader Prof. Yukihide Iwamoto at Kyushu University, succeeded by current representative Prof. Toshifumi Ozaki at Okayama University and secretary-general Prof. Kazuhiro Tanaka at Oita University, this network spearheads high-grade interventional registration trials. JCOG-BSTTSG conducts pivotal Phase II and randomized Phase III prospective trials to establish global standards of care for systemic and surgical management (Table 1), including:

- **JCOG0304:** Landmark study defining the efficacy and long-term outcomes of perioperative chemotherapy with doxorubicin and ifosfamide for high-grade extremity soft tissue sarcomas [96, 97].
- **JCOG1306:** Randomized Phase II/III trial comparing perioperative doxorubicin plus ifosfamide versus gemcitabine plus docetaxel for high-risk soft tissue sarcomas [98].
- **JCOG0905:** Landmark randomized Phase III trial evaluating intensified chemotherapy incorporating ifosfamide in poor responders to preoperative doxorubicin, cisplatin, and high-dose methotrexate (MAP) chemotherapy for newly diagnosed high-grade osteosarcoma [99, 100].
- **Ongoing frontier trials:** Modern protocols such as JCOG1802 (2ND-STEP study comparing second-line systemic agents for advanced soft tissue sarcoma) [101], 102, JCOG2102 (NACLESS study evaluating neoadjuvant chemotherapy versus immediate surgery for localized soft tissue sarcoma) [103], and JCOG2214INT (a randomized phase III international collaborative trial [STRASS 2] evaluating neoadjuvant chemotherapy for high-risk retroperitoneal sarcoma). Targeting resectable, high-risk dedifferentiated liposarcoma and leiomyosarcoma arising in the retroperitoneum, JCOG2214INT evaluates whether adding neoadjuvant chemotherapy (doxorubicin + ifosfamide or doxorubicin + dacarbazine) to standard curative surgery improves disease-free survival as the primary endpoint [104].

**Table 1:** Key prospective clinical trials conducted by the Japan Clinical Oncology Group Bone and Soft Tissue Tumor Study Group (JCOG-BSTTSG)

Trial ID/Name	Target population	Phase/Design	Treatment arms/Intervention	Key findings/Clinical significance	Reference(s)
JCOG0304	High-grade extremity soft tissue sarcoma (STS)	Phase II (Single-arm)	Perioperative chemotherapy with Doxorubicin + Ifosfamide (AI)	Established the safety and efficacy of perioperative AI chemotherapy as a standard regimen in Japan; achieved 2-year OS of 93.3%, 5-year OS of 85.0%, and 10-year OS of 82.5%, confirming excellent long-term survival.	[96, 97]
JCOG0905	Newly diagnosed high-grade osteosarcoma (poor responders to preoperative MAP)	Randomized Phase III	Postoperative MAP (Methotrexate, Doxorubicin, Cisplatin) vs. MAP + Ifosfamide (MAP+I)	Evaluated the efficacy of adding ifosfamide for poor histological responders (<90% tumor necrosis); no significant survival benefit was demonstrated (5-year EFS 58.0% for MAP vs. 55.1% for MAP+I, p=0.81; 5-year OS 72.8% vs. 69.4%, p=0.74).	[99, 100]
JCOG1306	High-risk localized non-uterine soft tissue sarcoma	Randomized Phase II/III	Perioperative Doxorubicin + Ifosfamide (AI) vs. Gemcitabine + Docetaxel (Gem/Doc)	Confirmed that standard AI chemotherapy remains superior to Gem/Doc in the first-line perioperative setting for high-risk STS.	[98]
JCOG1802 (2ND-STEP)	Advanced/relapsed/metastatic soft tissue sarcoma	Randomized Phase II	Head-to-head comparison of second-line systemic agents: Trabectedin vs. Eribulin vs. Pazopanib	Randomized trial establishing second-line treatment sequencing; results presented at ASCO 2025 demonstrated comparable efficacy across arms, with distinct subtype-specific activity and toxicity profiles.	[101, 102]
JCOG2102 (NACLESS)	Localized resectable high-risk soft tissue sarcoma	Randomized Phase III	Neoadjuvant + adjuvant chemotherapy vs. Immediate surgery + adjuvant chemotherapy	Ongoing trial investigating the true clinical value and timing of neoadjuvant chemotherapy compared with immediate curative surgery.	[103]
JCOG2214INT (STRASS 2)	Resectable high-risk retroperitoneal sarcoma (dedifferentiated liposarcoma & leiomyosarcoma)	Randomized Phase III (International collaborative)	Neoadjuvant chemotherapy (AI or Doxorubicin + Dacarbazine) followed by surgery vs. Surgery alone	International prospective collaborative study [STRASS2] evaluating whether neoadjuvant chemotherapy improves disease-free survival (DFS) in high-risk retroperitoneal sarcoma.	[104]

*AI* adriamycin (doxorubicin) and ifosfamide, *DFS* disease-free survival, *EFS* event-free survival, *MAP* methotrexate, adriamycin (doxorubicin), and cisplatin, *OS* overall survival, *STS* soft tissue sarcoma

#### Japan Ewing Sarcoma Study Group (JESS)

The Japan Ewing Sarcoma Study Group (JESS) was established in February 2003 as a multidisciplinary clinical trial group primarily composed of orthopaedic surgeons, paediatric oncologists, and radiation oncologists. Since the inauguration of the Japan Children's Cancer Group (JCCG) in 2014, JESS has functioned as a specialized committee within the JCCG infrastructure. From January 2005 to May 2008, JESS conducted the "Phase II Clinical Trial of Multidisciplinary Treatment for Localized Ewing Sarcoma Family of Tumors” (JESS04 Study) [105]. Enrolling 53 patients, the study achieved a three year disease-free survival (DFS) rate of 71.7% and a five year DFS rate of 69.6%—outcomes highly comparable to those reported by multi-institutional collaborative groups in Europe and North America. Subsequently, the JESS15 study was launched in 2016 and remains ongoing. Throughout this timeline, Prof. Toshifumi Ozaki and Prof. Kazuhiro Tanaka have served as committee chairs. Numerous orthopaedic surgeons have consistently driven the execution of these clinical trials, making profound contributions to the management of this rare, high-grade malignancy.

### Multidisciplinary synergy and the rise of JSTAR

Historically, orthopaedic surgeons in Japan served as the primary, comprehensive oncologists for sarcoma patients, directing not only surgical extirpation and reconstruction but also perioperative chemotherapy and disease surveillance. Analyses of national administrative and hospital-based cancer registries confirm that orthopaedic surgeons remain the backbone of sarcoma care, managing over 70% of primary bone sarcomas and approximately 40% of soft tissue sarcomas, as well as a substantial proportion of systemic therapies.

**Fig. 3:**
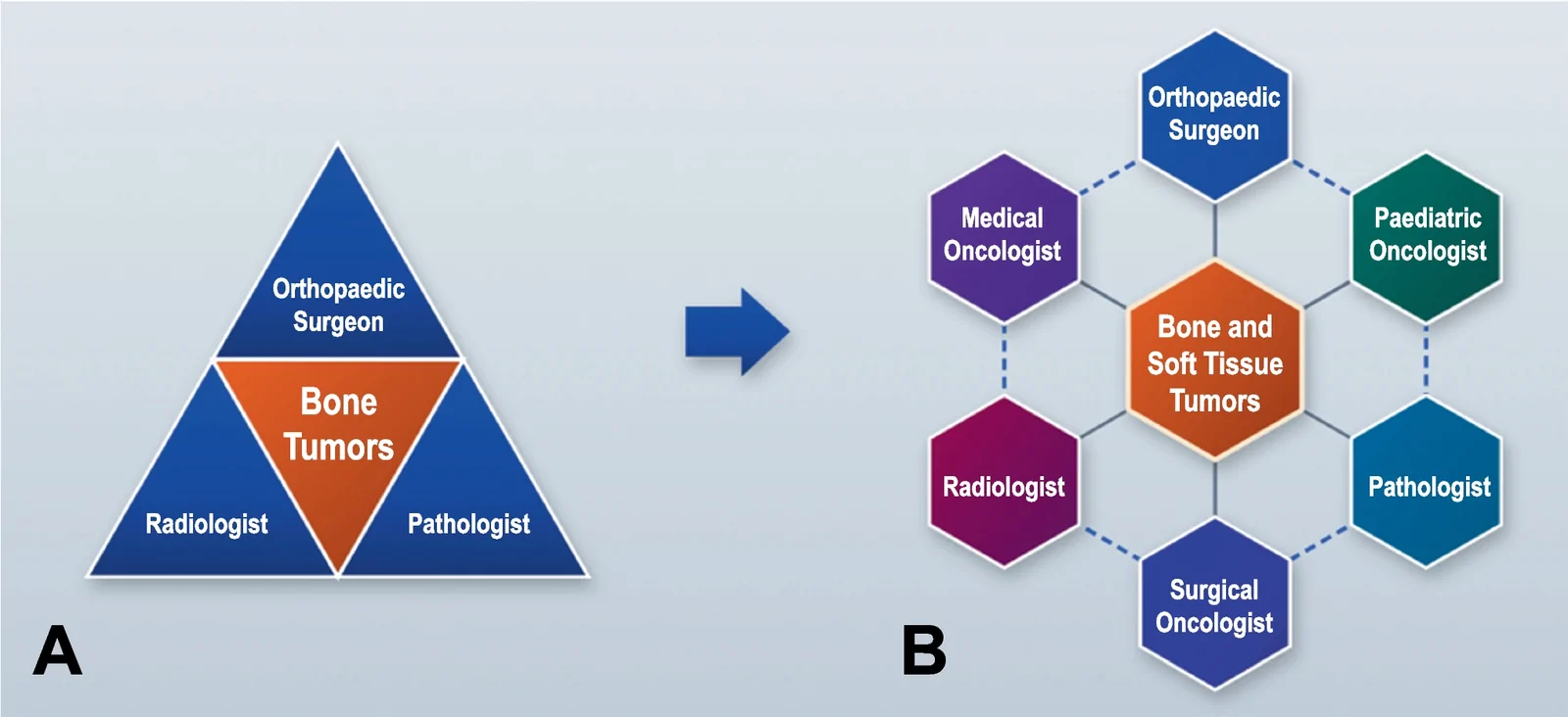
Conceptual shift in multidisciplinary sarcoma care networks. (Left) "Jaffe's Triangle": The classic triad model centered primarily on the Orthopaedic Surgeon, Radiologist, and Pathologist for primary bone tumour care. (Right) "JSTAR Constellation" (21st Century): The modern, expanded multidisciplinary platform represented by JSTAR, integrating orthopaedic surgeons, medical oncologists, pediatric oncologists, surgical oncologists, specialized nursing, allied health professionals, and patient advocacy groups around a central hub for bone and soft tissue tumours

However, while the classical 20th-century triad famously conceptualized by Henry L. Jaffe as "Jaffe’s triangle"—comprising the Orthopaedic Surgeon, Radiologist, and Pathologist—laid the foundational bedrock for team care [106], it has evolved to accommodate the complexity of modern oncology. With 30% to 60% of sarcoma cases affecting non-extremity regions (such as retroperitoneal, pelvic, and trunk malignancies) or requiring novel targeted therapies and immunotherapies, sarcoma management naturally shifted toward a 21st-century multidimensional, cross-disciplinary network. While the classical 20th-century triad was elegantly captured as 'Jaffe's Triangle', 21st-century sarcoma management has evolved into a dynamic 'JSTAR Constellation'—an expanded, multidimensional network uniting diverse specialties around the patient (Fig. 3).

In July 2017, through the leadership of Dr. Akira Kawai at the National Cancer Center Hospital and dedicated colleagues, this collaborative vision culminated in the founding of the Japanese Association of Sarcoma Treatment and Research (JSTAR) [107]. JSTAR was established as an all-inclusive, multi-professional academic society uniting orthopaedic surgeons, medical oncologists, paediatric oncologists, general and visceral surgeons, urologists, plastic surgeons, pathologists, radiologists, basic researchers, allied health professionals, and patient advocacy groups. In addition to hosting its Annual Meeting to promote cross-disciplinary academic exchange, JSTAR actively formulates cross-specialty clinical practice guidelines (such as the Retroperitoneal Sarcoma Clinical Practice Guidelines 2021) [108], conducts nationwide multidisciplinary clinical surveys, and fosters international collaborative networks. JSTAR places patient partnership at the core of its mission, working alongside patient advocacy groups to address unmet medical and societal needs.

To advance the quality of sarcoma care and research across Japan, JSTAR works in close, complementary partnership with the JOA. Functioning as "two sides of the same coin," the JOA and JSTAR operate in powerful synergy—harmonizing deep-rooted orthopaedic surgical tradition with broad multidisciplinary innovation, guideline standardization, and patient-centered advocacy.

## PILLAR 5: Onco-orthopaedics and future musculoskeletal care

As cancer survival rates improve worldwide, oncology increasingly manages cancer as a chronic condition, driving a crucial demand for functional preservation [109]. Japanese orthopaedic surgeons have led the conceptual development of Onco-Orthopaedics.

### The concept of "cancer locomo"

While traditional Orthopaedic Oncology focuses on primary bone and soft tissue sarcomas, Onco-Orthopaedics expands orthopaedic care to the comprehensive musculoskeletal management of all cancer patients. Pioneered by Prof. Hirotaka Kawano at Teikyo University, the JOA introduced the concept of "Cancer Locomo"—locomotive syndrome in cancer patients— defining it as mobility impairment directly or indirectly caused by cancer [110, 111]. Its etiology is classified into three distinct categories:

- **Type 1 (Locomotive dysfunction directly affected by cancer):** Bone metastases, pathological fractures, and spinal cord compression.
- **Type 2 (Locomotive dysfunction related to cancer treatment):** Muscle atrophy, sarcopenia, joint contractures, chemotherapy-induced peripheral neuropathy, and osteoporosis due to inactivity, surgery, systemic therapy, or radiation.
- **Type 3 (Locomotive dysfunction coexisting with cancer):** Pre-existing osteoarthritis or lumbar spinal stenosis that deteriorate during cancer treatment.

Because mobility loss caused by manageable musculoskeletal problems is often misattributed to systemic cancer progression, timely orthopaedic evaluation preserves Performance Status, enabling patients to safely continue primary cancer treatments and maintain their quality of life. Multidisciplinary bone metastasis boards across Japan now unite oncologists, orthopaedic surgeons, radiologists, palliative care specialists, and physical therapists to provide coordinated and proactive care [112]. As the most advanced super-aged society in the world, Japan is well positioned to generate new evidence and insights into the management of “Cancer Locomo.”

## Conclusion

Over the past century, Japanese orthopaedic oncology has evolved from an isolated surgical subspecialty into a multi-dimensional collaborative ecosystem. By combining quantitative surgical margin concepts, innovative recycled autograft techniques, translational therapeutic discoveries, nationwide prospective registries (JBSTTR), multi-institutional collaborations (JMOG, JCOG-BSTTSG, JESS), multidisciplinary platforms (JSTAR), and Onco-Orthopaedics, Japan offers a comprehensive, sustainable model for advancing orthopaedic oncology globally.

## Data Availability

No datasets were generated or analysed during the current study.

## Declarations

### AI-Assisted copy editing

During the preparation of this work, the authors used generative AI technology (Gemini, Google) strictly for AI-assisted copy editing (improving readability, stylistic polish, and ensuring correct English grammar and formatting). All substantive content, conceptual ideas, literature selection, and generative editorial work were originated and critically verified by the authors, who take full human accountability for the final manuscript.
